# Simultaneous Quantification of *Vibrio metoecus* and *Vibrio cholerae* with Its O1 Serogroup and Toxigenic Subpopulations in Environmental Reservoirs

**DOI:** 10.3390/pathogens9121053

**Published:** 2020-12-16

**Authors:** Tania Nasreen, Nora A. S. Hussain, Mohammad Tarequl Islam, Fabini D. Orata, Paul C. Kirchberger, Rebecca J. Case, Munirul Alam, Stephanie K. Yanow, Yann F. Boucher

**Affiliations:** 1 Department of Biological Sciences, University of Alberta, Edmonton, AB T6G 2E9, Canada; tnasreen@ualberta.ca (T.N.); nahussai@ualberta.ca (N.A.S.H.); mislam@ualberta.ca (M.T.I.); orata@ualberta.ca (F.D.O.); rj.case@ntu.edu.sg (R.J.C.); 2 Department of Integrative Biology, University of Texas at Austin, Austin, TX 78712, USA; pkirchberger@utexas.edu; 3 Singapore Centre for Environmental Life Sciences Engineering (SCELSE), Nanyang Technological University, Singapore 637551, Singapore; 4 School of Biological Sciences, Nanyang Technological University, Singapore 637551, Singapore; 5 Centre for Communicable Diseases, International Centre for Diarrhoeal Disease Research, Bangladesh (ICDDR, B), Dhaka 1000, Bangladesh; munirul@icddrb.org; 6 School of Public Health, University of Alberta, Edmonton, AB T6G 1C9, Canada; yanow@ualberta.ca; 7 Department of Medical Microbiology and Immunology, University of Alberta, Edmonton, AB T6G 2E1, Canada; 8 Singapore Centre for Environmental Life Sciences Engineering (SCELSE), National University of Singapore, Singapore 637551, Singapore; 9 Saw Swee Hock School of Public Health, National University of Singapore, Singapore 117549, Singapore

**Keywords:** *Vibrio cholerae*, *Vibrio metoecus*, qPCR, serogroup, toxigenic and non-toxigenic, O1, cholera-endemic

## Abstract

*Vibrio metoecus* is a recently described aquatic bacterium and opportunistic pathogen, closely related to and often coexisting with *Vibrio cholerae.* To study the relative abundance and population dynamics of both species in aquatic environments of cholera-endemic and cholera-free regions, we developed a multiplex qPCR assay allowing simultaneous quantification of total *V. metoecus* and *V. cholerae* (including toxigenic and O1 serogroup) cells. The presence of *V. metoecus* was restricted to samples from regions that are not endemic for cholera, where it was found at 20% of the abundance of *V. cholerae*. In this environment, non-toxigenic O1 serogroup *V. cholerae* represents almost one-fifth of the total *V. cholerae* population. In contrast, toxigenic O1 serogroup *V. cholerae* was also present in low abundance on the coast of cholera-endemic regions, but sustained in relatively high proportions throughout the year in inland waters. The majority of cells from both *Vibrio* species were recovered from particles rather than free-living, indicating a potential preference for attached versus planktonic lifestyles. This research further elucidates the population dynamics underpinning *V. cholerae* and its closest relative in cholera-endemic and non-endemic regions through culture-independent quantification from environmental samples.

## 1. Introduction

*Vibrio cholerae* is an autochthonous aquatic bacterium [1] which shows variable physiologies, from non-pathogenic to extremely virulent strains capable of causing a life-threatening diarrheal infection: cholera [2]. According to the World Health Organization (2016), every year, 1.4 to 4 million people are infected with cholera, and 21,000 to 143,000 people die from this disease [3]. This species comprises over 200 serogroups [4], but strains of the O1 and O139 serogroups are distinguished as the most virulent, having caused some of the most devastating pandemics in human history [5,6,7,8]. Other serogroups are collectively known as non-O1/non-O139 and can cause sporadic outbreaks of diarrheal disease less severe than cholera [9]. *V. cholerae* O139 was only found to be associated with isolated cholera cases after two epidemics occurred in 1993 and in 2002 [10,11]. During 2005, the O139 serogroup was isolated sporadically from both clinical and environmental samples in Bangladesh but there was no reported large-scale outbreak of cholera caused by this serogroup. Further studies confirmed that four *V. cholerae* isolated from cholera patients identified as *V. cholerae* O139 during 2013 and 2014 in Bangladesh by typing and whole genome sequencing [2,12,13]. However, *V. cholerae* O1 has been prevalent in the Ganges Delta for several hundred years; this remains the only place in the world where cholera has been continually endemic since the first modern pandemic in 1817 [14,15]. Much research has been done to characterize the physiology of pathogenic *V. cholerae* strains, but still, we have limited understanding of the variation of *V. cholerae* population composition and abundance over time and between areas, which influences where and when epidemics are likely to occur.

*Vibrio metoecus* is a recently described species, which has been co-isolated with *V. cholerae* from aquatic environments in the USA East coast [16,17,18]. Based on biochemical, genotypic and phylogenetic evidence, *V. metoecus* is the closest known relative of *V. cholerae* [16]. It has been recovered not only from the environment but also from a variety of human specimens (blood, stool, ear and leg wounds) in opportunistic infections across the USA (CDC, Atlanta, GA, USA) [16]. Currently, no studies have assessed the abundance of *V. metoecus* in aquatic habitats. Information on the distribution of *V. metoecus* in its natural reservoirs is essential not only to provide insight into transmission routes from the environment to humans, but also because of its co-occurrence with *V. cholerae* and the possibility of horizontal gene transfer (HGT) between the species, which could lead to the evolution of more virulent strains of *V. metoecus* [19].

Most surveys of *V. cholerae* have used culture-based methods, requiring significant time and effort, and their reliance on selective enrichment prevents any form of absolute quantification [20]. Moreover, these culture-based techniques are unable to detect the viable but non-culturable (VBNC) form of *V. cholerae* [21,22,23] and thus underestimate the abundance of toxigenic and non-toxigenic *V. cholerae* in the environment [24]. To circumvent this problem, several studies have used antibody or hybridization-based molecular detection of toxigenic *V. cholerae* from clinical [25,26] and environmental samples [20,27,28,29,30], but most are qualitative, and none have been field-tested to comprehensively survey its abundance in natural environments [31,32]. Another molecular tool, real-time quantitative PCR (qPCR), has been used to measure both the presence and abundance of target species [33,34]. This technique can estimate the presence of as few as three bacterial genome equivalents in a sample and provides the high throughput required to investigate the ecological distribution of the organism [35]. Several gene markers have been developed for the detection of the *V. cholerae* species by qPCR, including the *ompW* gene encoding the outer membrane protein [36], the *hlyA* gene encoding for hemolysin [36,37] and *gbpA* gene encoding the N-acetyl glucosamine-binding protein A [38], and the *rtx* gene cluster encoding the repeats in toxin protein [39]. During the assessment of different primers used in previous studies, it was found that the commonly used primers designed for *ompW* also amplified that gene from *V. metoecus* (in this study) and *Vibrio mimicus* [40]. Copy number is also a confounding factor, as *rtx* [39], *hlyA* and *ctxA* can be present in multiple copies in *V. cholerae* genomes [41,42]. Although *gbpA* is present as a single copy, it can also be found in environmental strains belonging to other *Vibrio* species including *Vibrio alginolyticus*, *Vibrio metschnikovii*, *Vibrio mimicus*, *Vibrio vulnificus* and *Vibrio parahaemolyticus* [43]. Lack of species specificity and the presence of multiple copies in single cells make quantitative estimates unreliable. Furthermore, given that a single lineage of *V. cholerae* is responsible for all major outbreaks [phylocore genome/pandemic generating (PG) *V. cholerae*] [8,44] (Supplementary Figure S1), detection of other, mostly harmless, genotypes can be misleading in evaluating the risk of outbreaks. Attempts to accurately quantify toxigenic *V. cholerae* using the cholera toxin (CT) gene *ctxA* have been made; while the reference El Tor strain N16961 carries a single copy of this gene, other *V. cholerae* strains carry several copies of this element and it is also occasionally found in other *Vibrio* species [41,45,46,47]. The *rfbO1* gene targeting *V. cholerae* strains carrying the O1 antigens has also been used to detect pandemic *V. cholerae* [48,49]. Although it is a single copy gene, many strains unrelated to this lineage can also carry it [50].

To overcome the limitations of currently used molecular markers, we developed a multiplex qPCR assay for simultaneous detection and quantification of *V. cholerae* O1 (*rfbO1*)*,* toxigenic *V. cholerae* (*ctxA*), total *V. cholerae* (*viuB*) and *V. metoecus* (*mcp).* This optimized qPCR technique allows the quantitative study of *V. cholerae* populations directly from DNA extracted from aquatic biomass, without the need for cultivation. This overcomes the problem that many *V. cholerae* cells in water are viable but not culturable (VBNC) [27], which has so far made it difficult to the study this organism in the environment. Quantifying the presence of *ctxA* and *rfbO1* simultaneously resolves drawbacks specific to the separate use of these two gene markers. Overestimation of the number of toxigenic cells because of the presence of multiple copies of *ctxA* in single cells is detected as discrepancies with *rfbO1* abundance. Likewise, overestimation of toxigenic O1 cells due to the presence of non-toxigenic O1 strains is identified through discrepancies with *ctxA* counts.

Furthermore, by measuring the relative abundance of toxigenic and O1 serogroup strains in the total *V. cholerae* population as well as *V. metoecus* abundance, this assay provides information about intraspecies and interspecies population dynamics, which could yield insights into variations in the natural abundance of toxigenic strains. It is also useful to look at the total *V. cholerae* population, as some strains lacking the two main virulence factors for cholera—CT and the toxin-coregulated pilus (TCP)—can still be pathogenic to humans [51]. This assay has both a low detection limit (three copies per reaction) and higher specificity than comparable assays. Besides being the first assay to allow molecular quantification of *V. metoecus*, it is also the first one to extensively test environmental samples for *V. cholerae* by measuring the absolute abundance of the species and its O1 serogroup and toxigenic subpopulations in areas that are endemic for cholera.

## 2. Results and Discussion

### 2.1. A Specific and Efficient Multiplex qPCR Assay to Detect V. cholerae and V. metoecus

**Figure 1:**
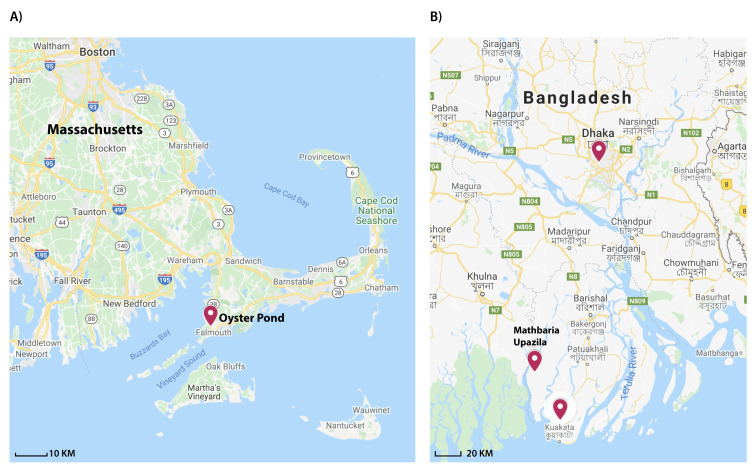
Sampling sites for environmental water samples collected to evaluate the qPCR assay developed in this study. (**A**) Oyster Pond (Falmouth, MA, USA) a non-endemic site for cholera. (**B**) Map of Bangladesh, identifying the two coastal regions (Kuakata and Mathbaria) and inland region (Dhaka) where samples were collected.

**Figure 2:**
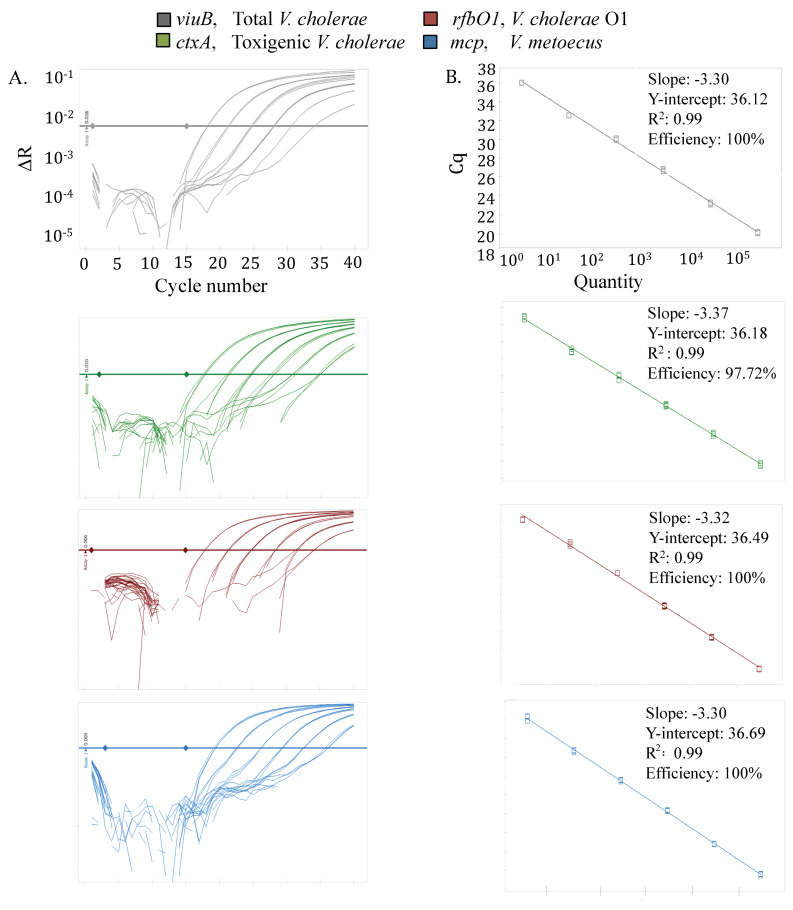
Multiplex real-time qPCR for simultaneous detection and quantification of *V. cholerae* and *V. metoecus*. Four gene markers with fluorogenic probes were used: *viuB* (*V. cholerae* specific), *ctxA* (toxigenic *V. cholerae* specific), *rfbO1* (*V. cholerae* O1 specific) and *mcp* (*V. metoecus* specific). Template DNA was purified from reference cultures (*V. cholerae* N16961 and *V. metoecus* RC341) and was serially diluted in 10-fold increments to yield concentration ranging from 3 × 10^6^ to 3 copies per reaction (from left to right). Fluorescence was measured in relative units. The panel (**A**) illustrates amplification curves and the panel (**B**) shows their corresponding standard curves. Each reaction was done in triplicate.

We developed a specific and sensitive qPCR method for the quantification of total *V. cholerae* and *V. metoecus*, as well as toxigenic and O1 serogroup *V. cholerae*, all sampled from their natural aquatic environments (Figure 1). Primers and probes developed for all four marker genes displayed 100% specificity (Table 1) using 50 bacterial strains, including 27 *V. cholerae* strains of various serogroups and toxigenic potential, as well as 18 *V. metoecus* and closely related *Vibrio* species. This assay was more specific than any previously published, with no cross-amplification between *V. cholerae* and other bacteria, including its closest relative, *V. metoecus*. The efficiency of each assay was 95% to 100% (R^2^ value 0.99 and slope—3.3 ± 0.07) (Figure 2).

The *viuB* and *mcp* markers are specific to *V. cholerae* and *V. metoecus* and present in single copies, facilitating quantification of the absolute abundance of these two species. Additionally, the *ctxA* and *rfbO1* markers are specific for detection of toxigenic and O1 serogroup strains, respectively. Previous qPCR studies often took advantage of these two genes but did not combine them, resulting in missed information that was required to correlate toxigenic *V. cholerae* and strains representing the PG lineage [30,36]. Although the region selected for *ctxA* gene amplification to indicate the presence of toxigenic *V. cholerae* is specific to this particular species [52], the presence of *ctxAB* has been reported in CTXφ phages present in aquatic environment, which can be the source of *ctxA* positive results [53]. Multiple copies of *ctxA* can also be present in certain *V. cholerae* genomes, biasing quantification results [47,54]. The simultaneous amplification of the single copy *rfbO1* specific for *V. cholerae* O1 strains compensates for these flaws, as the vast majority of cholera cases are caused by CTX positive strains of the O1 serogroup. The co-occurrence of these two markers at similar levels allows accurate quantification of toxigenic O1 strains.

There are many rapid assays already being used to detect certain serotypes of *V. cholerae*, but most of them are confined to qualitative detection and do not provide any data on the abundance of this organism in the environment [52,55,56,57]. A few available assays are quantitative, but the limitations in sensitivity are >5 to 50 gene copies per reaction [38,58], and they detect no more than two marker genes, or lack specificity [32]. For example, a real-time PCR assay using four different genetic markers i.e., *rtxA*, *epsM*, *ompW* and *tcpA* to detect *V. cholerae* also detected *V. mimicus* non-specifically by amplification of *ompW* [32]. Other genes currently used for detection and/or quantification of *V. cholerae* such as *hlyA*, *zot*, *ompU*, *toxR* and *groEl*, also suffer from a lack of species specificity [52,55,59]. Many of these genes share sequence similarity with homologs in closely related species or can be present in multiple copies in *Vibrio* genomes, and therefore can pose problems for specific detection and quantification. Moreover, there is no published assay that detects and quantifies the abundance of *V. metoecus* along with *V. cholerae*. Most assays have been evaluated by artificially spiking water samples with known concentrations of laboratory strains, and none has been directly applied to a significant number of environmental samples in a region that is endemic for cholera.

**Figure 3:**
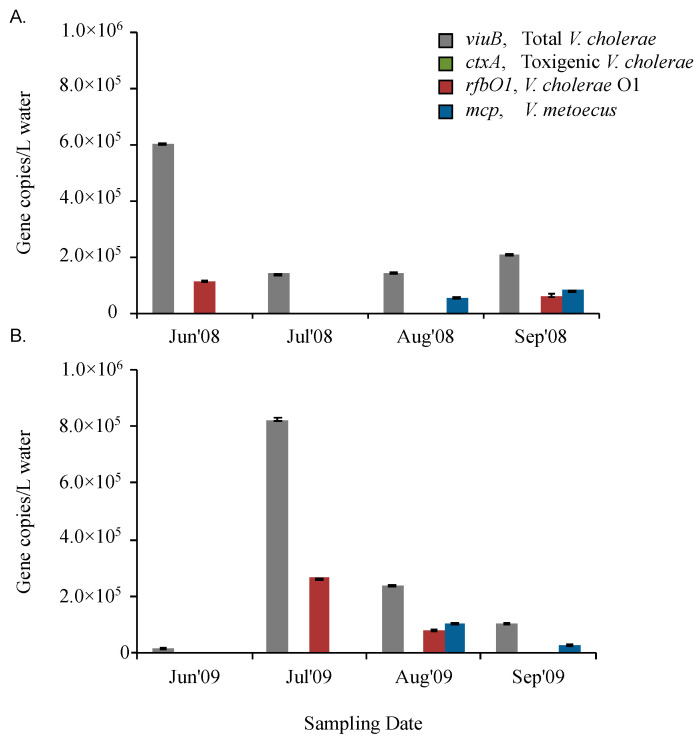
Temporal variation of abundance for *V. cholerae* along with its toxigenic and serogroup O1 subpopulations and its close relative *V. metoecus* in Oyster Pond, MA, USA. Environmental water samples collected during the months of June to August in two successive years: 2008 (**A**) and 2009 (**B**), were analyzed using the developed qPCR assay. The *viuB* gene was used to quantify total *V. cholerae*; *ctxA* and *rfbO1* were used to measure toxigenic *V. cholerae* and *V. cholerae* O1, respectively; the abundance of *V. metoecus* was estimated using the *mcp* gene. All four genes were tested for each sample and the absence of a bar in the graph denotes that the target gene was absent or present below the detection limit of the assay. Each qPCR reaction was run in triplicate. Mean values are shown with error bars indicating the standard deviation between three technical replicates. *ctxA* could not be detected at any time sampled at this site.

**Figure 4:**
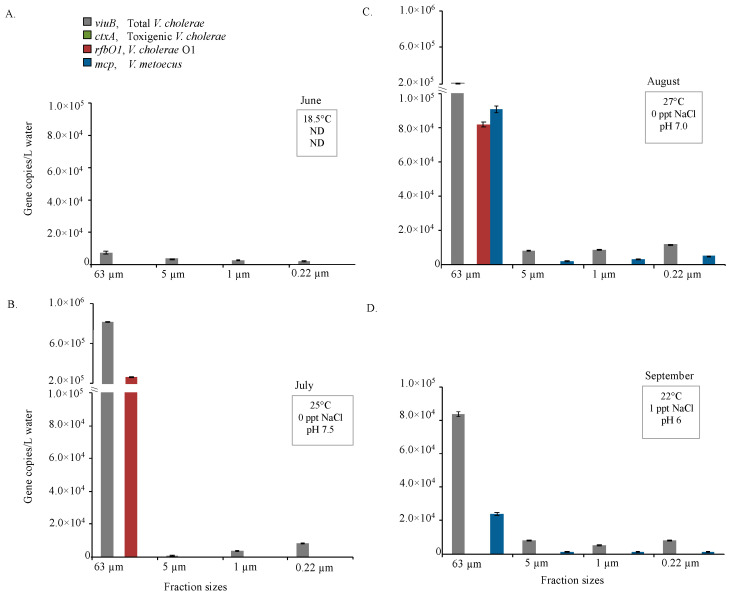
Distribution in different water fraction sizes of *V. cholerae* along with its toxigenic and serogroup O1 subpopulations and its close relative *V. metoecus* in Oyster Pond, MA, USA. Environmental water samples were collected during the months of June (**A**), July (**B**) August (**C**) and September (**D**) in 2009. These samples were fractionated by size through sequential filtration and bacteria were quantified by qPCR of marker genes on DNA extracted from the filters. The *viuB* gene was used to quantify total *V. cholerae*; *ctxA* and *rfbO1* were used to measure toxigenic *V. cholerae* and *V. cholerae* O1, respectively; the abundance of *V. metoecus* was quantified using the *mcp* gene. Each qPCR reaction was run in triplicate. Mean values are shown with error bars indicating the standard deviation between technical replicates. Temperature, pH and salinity of water collected each month are shown in boxes on the upper right corner of each graph. ND indicates not done.

**Figure 5:**
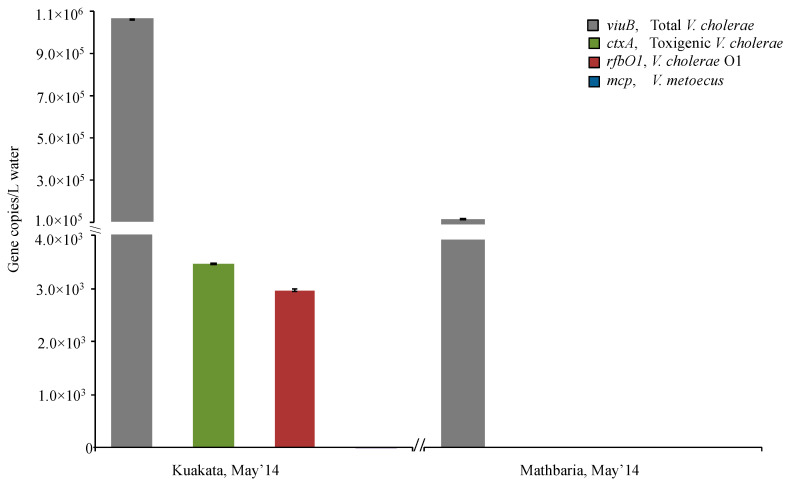
Abundance of *V. cholerae* along with its toxigenic and serogroup O1 subpopulations and its close relative *V. metoecus* in two different coastal regions in Bangladesh. Environmental water samples were collected from Kuakata and Mathbaria during the month of May in 2014 and bacteria were quantified by qPCR of marker genes. The *viuB* gene was used to quantify total *V. cholerae*; *ctxA* and *rfbO1* were used to measure toxigenic *V. cholerae* and *V. cholerae* O1, respectively; the abundance of *V. metoecus* was quantified using the *mcp* gene. Each qPCR reaction was run in triplicate. Mean values are shown with error bars indicating the standard deviation between technical replicates.

**Figure 6:**
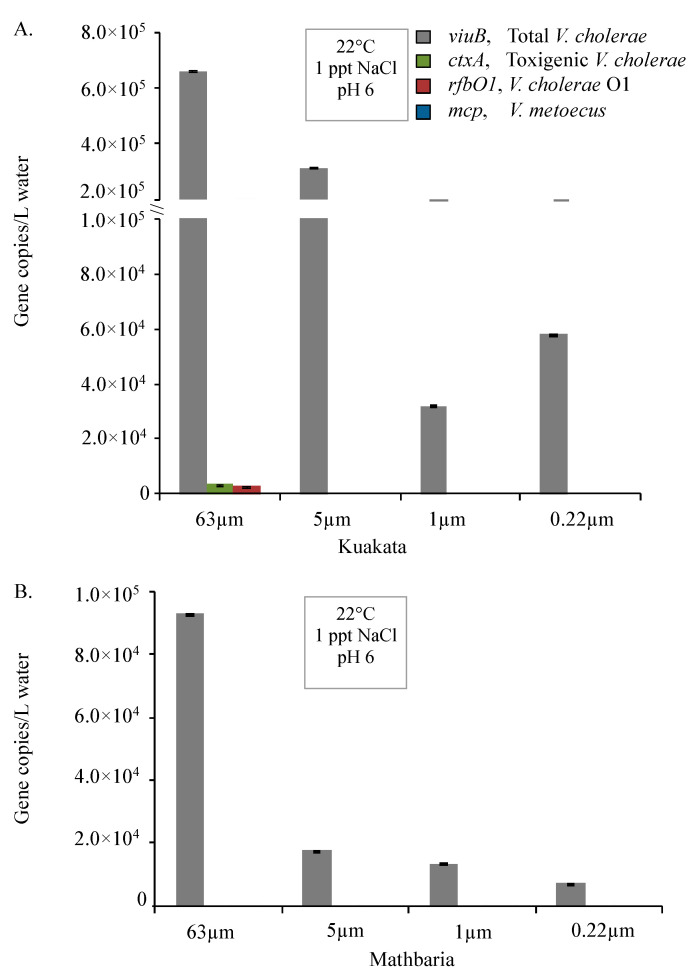
Abundance of *V. cholerae* along with its toxigenic and serogroup O1 subpopulations and its close relative *V. metoecus* in two different coastal regions in Bangladesh. Environmental water samples were collected from Kuakata (**A**) and Mathbaria (**B**) during the month of May in 2014 and bacteria were quantified by qPCR of marker genes. The *viuB* gene was used to quantify total *V. cholerae*; *ctxA* and *rfbO1* were used to measure toxigenic *V. cholerae* and *V. cholerae* O1, respectively; the abundance of *V. metoecus* was quantified using the *mcp* gene. Each qPCR reaction was run in triplicate. Mean values are shown with error bars indicating the standard deviation between technical replicates.

**Figure 7:**
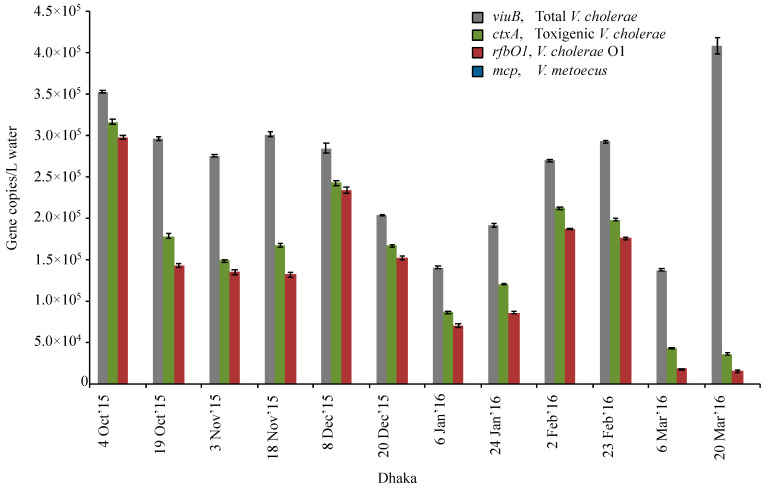
Temporal variation of abundance for *V. cholerae* along with its toxigenic and serogroup O1 subpopulations and its close relative *V. metoecus* in a central region of Bangladesh (Dhaka). Environmental water samples were collected bi-weekly from the months of October 2015 to March 2016 and bacteria were quantified by qPCR of marker genes. The *viuB* gene was used to quantify total *V. cholerae*; *ctxA* and *rfbO1* were used to measure toxigenic *V. cholerae* and *V. cholerae* O1, respectively; the abundance of *V. metoecus* was quantified using the *mcp* gene. Each qPCR reaction was run in triplicate. Mean values are shown with error bars indicating the standard deviation between technical replicates.

It is important for any environmental application to determine the sample limit of detection. This value represents the lowest quantity of the target DNA that can be reliably detected and quantified in a certain volume of sample at a probability level of 95% [60]. In this study, we found the analytical detection limit for all four gene markers to be three copies per reaction (Figure 2), with 1.5 × 10^6^ copies/L of water as the lowest detectable number without filtration, which is comparable to previous studies [59]. Concentrating samples by filtration of large volumes of water (0.05 L to 10 L) made it possible to lower the detection limit. This limit was 9.0 × 10^2^ copies/L for Oyster Pond (USA) samples after filtering 566 mL of water (Figure 3 and Figure 4B). The lowest detection limits after filtration of 3.0 × 10^3^ copies/L (from 10 L of water) (Figure 5 and Figure 6A) and 1.5 × 10^4^ copies/L (from 50 mL of water) (Figure 7) were determined for samples from the Kuakata and Dhaka (Bangladesh) sites, respectively.

The sensitivity of PCR-based assays for quantifying *V. cholerae* from environmental samples is highly dependent on efficient DNA extraction and removal of potential inhibitors. All the environmental DNA samples were treated with One step PCR inhibitory removal kit after DNA extraction to reduce residual inhibitors. Samples were assessed in ten-fold dilutions (1/10) to determine the effect of inhibitors in the assay. The 10× dilution of samples shifted the Cq values by 3.3 cycles ± 0.05 (Supplementary Figure S2), which is consistent with no significant inhibition.

### 2.2. V. cholerae and V. metoecus Co-Occur Seasonally in a Temperate Coastal Location

With an optimized multiplex qPCR assay, it was possible for the first time to determine the abundance of *V. cholerae* and *V. metoecus* simultaneously in water reservoirs in a cholera non-endemic area (USA). *V. cholerae* is an ubiquitous member of bacterial communities in temperate and tropical aquatic environments around the world [5] whereas *V. metoecus* is a recently described species, which has been co-isolated with *V. cholerae* from the USA East Coast aquatic environment and associated with eel fish in Spain, as well as clinical cases from around the USA [16,61,62,63].

To compare the qPCR assay to culture-dependent methods of detection, it was applied to samples from which *V. cholerae* and *V. metoecus* had been isolated by cultivation and samples from which no organisms had been isolated (Table 2). These water samples were collected in summer (June to September) over two successive years (2008 and 2009) from Oyster Pond in Falmouth (MA) on the East Coast of the USA. Using our novel multiplex assay on DNA extracted from biomass of the same samples, we found the abundance of *V. cholerae* was 1.4 × 10^5^ to 8.2 × 10^5^ copies/L in Oyster Pond from June to September in 2008 and 2009 (Supplementary Figure S3). Strikingly, *V. metoecus* was only detected at the end of the season (the months of August and September) in both years, at abundances of approximately 5.6 × 10^4^ to 1.0 × 10^5^ copies/L (Figure 3). *V. cholerae* was consistently more abundant than *V. metoecus* and present throughout the summer. Using a culture-based approach, it has been suggested that *V. cholerae* is ten times more abundant than *V. metoecus* at that particular location [62]. Based on the qPCR approach used here, *V. cholerae* was approximately three times more abundant than *V. metoecus*, suggesting that the former is more readily culturable than the latter (Figure 3). Moreover, *V. metoecus* was neither isolated by conventional culture method nor detected by qPCR in Bangladesh, suggesting a different geographical distribution than *V. cholerae* (Figure 5).

### 2.3. O1 Serogroup Strains Are Important Members of a Temperate Coastal V. cholerae Population

The presence/absence of toxigenic or O1 serogroup *V. cholerae* was determined using two sets of primers targeting *ctxA* and *rfbO1*. There was no amplification of *ctxA* in the Oyster Pond samples, but *rfbO1* was present sporadically: 1.1 × 10^5^ copies/L were present in June 2008 and 6.2 × 10^4^/L in September 2008 (Figure 3), while 2.6 × 10^5^ copies/L *rfbO1* were detected in July 2009 and 8.2 × 10^4^ copies/L in August of 2009 (Figure 3). The proportion of *rfbO1* positive *V. cholerae* was, on average, ~18% of total *V. cholerae* in Oyster pond over these periods of sampling, ranging from 15% to 21% in individual samples. O1 serogroup of *V. cholerae* were consistently found during the four warmest months (June to September) of 2008 and 2009 (Figure 3). Interestingly, in water samples that were fractionated by size (2009), *rfbO1* was detected only in the largest size fraction (>63 μm) (Figure 4).

No O1 serogroup strains could be obtained from Oyster pond samples by conventional culture methods, despite the isolation of over 385 *V. cholerae* strains [62] (Table 2). *V. cholerae* enters into VBNC form due to environmental stress condition, where cells remain alive but cannot be revived using standard cultivation methods [64]. During inter-epidemic periods, toxigenic *V. cholerae* are believed to be maintained in low numbers, attaching to particles in a state of VBNC [65].

The inability to isolate *V. cholerae* O1 in most of the samples indicates that culture-based methods could lead to an underestimation of the occurrence of *V. cholerae* O1 in environmental reservoirs due to their low abundance or VBNC state [36]. The inability to isolate *V. cholerae* O1 strains in 2009 samples collected from Oyster pond together with their detection at a relatively high abundance (Table 2, Figure 3) by qPCR likely indicates the presence of VBNC cells, questioning the assumption that *V. cholerae* O1 is rare in cholera-free regions [66].

### 2.4. Toxigenic O1 Serogroup Strains Are Constantly Present at Dangerous Levels in Dhaka Freshwater

In Bangladesh, data on the incidence of cholera show that the disease occurs year round in the Ganges delta region of Bangladesh with seasonal peaks typically before (March to May) and after (September to December) monsoon [67,68]. However, using culture-based methods, direct isolation of toxigenic *V. cholerae* O1 from aquatic reservoirs was not readily possible even during the peak season unless further enrichment in an alkaline peptone water medium was performed [28]. To determine if culture-based monitoring underestimates the presence of toxigenic *V. cholerae* O1, bi-weekly sampling was done over six consecutive months in the Rampura area of Dhaka city in Bangladesh.

Biomass was collected from water samples by filtration on 0.22 μm membranes with no size fractionation. The abundance of the *viuB* marker gene, which corresponds to the total *V. cholerae* population, ranged from 1.3 × 10^5^ to 4.0 × 10^5^ copies/L from October 2015 to March 2016 (Figure 7). The *ctxA* gene used to track toxigenic *V. cholerae* was found at 3.6 × 10^4^ copies/L to 3.2 × 10^5^ copies/L and the *rfbO1* gene used to track *V. cholerae* O1 was detected at similar levels, ranging from 1.5 × 10^4^ copies/L to 2.9 × 10^5^ copies/L, corresponding to 50–60% of the total *V. cholerae* population (Figure 7). There was consistent occurrence of *V. cholerae* and its toxigenic and serogroup O1 subpopulations at similar abundances throughout the six months of sampling, suggesting that both genes were found in the same cells, which is typical of 7th pandemic *V. cholerae* El tor strains that are currently responsible for most cholera cases in Bangladesh [8]. Conventional culture methods only recovered *V. cholerae* O1 from October 2015, and *V. cholerae* non-O1 was isolated from each of these six months (Table 2).

Unexpectedly, analysis of water samples from the Rampura area (Dhaka, Bangladesh) (Figure 1) revealed persistent toxigenic *V. cholerae* O1 at a low abundance (1.3 × 10^5^ to 4.0 × 10^5^ copies/L), but a high proportion of the total *V. cholerae* population (up to 84%) (Figure 7). With the infectious dose of *V. cholerae* being in the order of 10^3^ to 10^8^ bacterial cells [69]; the water in this region is a permanent reservoir of toxigenic *V. cholerae* that can readily cause potential outbreaks when ingested with contaminated water or food. Water bodies around Dhaka city are surrounded by a dense human population that extensively interacts with them, potentially resulting in the circulation of pathogenic *V. cholerae* in that particular environment, even outside of periods in which cholera cases are frequent. This stands in contrast with the megacity experiencing two seasonal outbreaks of cholera before the monsoon in March to May and just after the monsoon in September to November [70]. The lack of correlation between toxigenic *Vibrio cholerae* in water reservoirs and the number of cholera cases in Dhaka remains to be elucidated: both public health (such as changes in drinking water sources or flooding) and biological (seasonal change in virulence levels) explanations for this discrepancy should be explored.

Another interesting observation at the inland Dhaka site was that the number of *ctxA* gene copies detected was always slightly higher than *rfbO1* (~20%). It is known from previous research that El Tor strain N16961 carries a single copy of the cholera toxin prophage whereas there could be variation in copy number in other El Tor *V. cholerae* strains arising from selective pressure [8,47,71,72]. Also, the presence of CTX phages in the aquatic environment may significantly impact the abundance of *ctxA* positive cells in the environment.

In contrast to the inland Dhaka site, the O1 serogroup was only detected at low abundance or was undetectable at the two coastal sites sampled in Bangladesh. *V. cholerae* O1 was found at very low abundance in Kuakata (May 2014) and absent from Mathbaria during the single month sampled (Figure 5, Figure 6), whereas in a previous study, detection of toxigenic *V. cholerae* O1 by DFA (direct fluorescent antibody) in water samples collected bi-weekly from March to December 2004 from six ponds in Mathbaria fluctuated from <10 to 3.4 × 10^7^ CFU/L [67]. It is noteworthy that during the period of this latter study, many cholera cases were recorded in Mathbaria, while they were much rarer in 2014. In the same 2004 study, no strains belonging to the O1 serogroup were found among the six hundred strains isolated from coastal areas in Bangladesh, confirming our observations in an area (Oyster pond, MA, USA) non-endemic for cholera that *V. cholerae* O1 is challenging to isolate (Table 2). The constant presence of *V. cholerae* O1 in Dhaka as opposed to a more stochastic appearance in coastal locations suggests that the level of human interaction with water bodies influences its population dynamic.

Higher abundance of *V. cholerae* and *V. metoecus* in larger size fractions indicates preference for particle association. Amongst the samples collected from endemic (Bangladesh) and non-endemic (Oyster Pond, MA, USA) regions, overall counts of *V. cholerae* ranged from 1 × 10^4^ copies/L to 1 × 10^6^ copies/L for each sample (Figure 3, Figure 5 and Figure 7). In Oyster pond, most of *V. cholerae* (~93%), including ~18% of the O1 serogroup *V. cholerae*, were detected in the largest fraction size (>63 μm). Similarly, the majority of *V. cholerae* cells from coastal areas of Bangladesh (~62%) were detected in the largest fraction size (> 63 μm) (Figure 5 and Figure 6). The toxigenic *V. cholerae ctxA* marker gene and O1 serogroup strains *rfbO1* gene were found only in the >63 μm size fraction and at very low abundance in Kuakata (3.4 × 10^3^ and 2.9 × 10^3^ copies/L, respectively) (Figure 6).

This skewed distribution of *V. cholerae* toward the largest fraction size (Figure 4 and Figure 6) suggests that most cells are associated with large particles, zooplankton, or phytoplankton hosts in environmental reservoirs [6,8,53,73]. This explains why in rural areas of Bangladesh, filtering environmental water through folded cloth facilitates the reduction of particles and debris and significantly lowers the risk of cholera [74].

## 3. Materials and Methods

### 3.1. Bacterial Cultures and DNA Template Preparation

Different isolates of *Vibrio* spp that were used to validate primer specificity and assay sensitivity in this study are listed in Table 1 and Supplementary Table S1. All *Vibrio* strains were grown in tryptic soy broth (TSB) (Becton Dickinson, Franklin Lakes, NJ, USA) with 1.0% NaCl (BDH), incubated at 30 °C and 200 rpm. For non-*Vibrio* strains (Supplementary Table S1), TSB without 1.0% NaCl was used under the same growth conditions. Genomic DNA was extracted from overnight cultures using the DNeasy Blood and Tissue Kit (QIAGEN, Hilden, Germany) and quantified using the Quant-iT PicoGreen dsDNA Assay Kit (Molecular Probes, Eugene, OR, USA) with a Synergy H1 microplate reader (BioTek, Winooski, VT, USA).

### 3.2. DNA Extraction from Biomass and Isolation of Organisms from Environmental Water Samples

Water samples were collected from Bangladesh (a cholera-endemic region) and the USA East Coast (a cholera-free region) (Figure 1) at different time points. Environmental water samples from Oyster Pond (Falmouth, MA, USA) were collected during the months of June to September, 2008 and 2009, as previously described [62]. Briefly, triplicate samples were obtained at a 0.5 m depth and a distance of 5 m from each other. Samples from 2009 were size fractionated, where ten litres of water were first filtered through a 63μm nylon mesh net to capture large particles such as zooplankton. Large particles were crushed in a 50 mL tissue grinder after transfer using 20 mL of sterile filtered local water. Two milliliters of crushed material (equivalent to one litre of water) was resuspended in 48 mL of sterile filtered local water and pushed through a 4.5 cm Millipore Durapore filter (0.22 μm pore size) using a polypropylene syringe.

Similarly, one litre of water passed through the mesh net was pushed through a series of in-line 4.5 cm Millipore Durapore filters (sizes 5 μm, 1 μm and 0.22 μm) using a peristaltic pump. All disposable equipment was sterile, and all filter casing and tubing was sterilized before sampling. DNA extraction from the filters using a QIAGEN DNeasy Blood and Tissue Kit was performed as follows: 0.25 g of sterile zirconium beads were added to cut-up filter pieces in a 1.5 mL screw cap tube with 360 μL Cell Lysis Buffer ATL, and bead beating was performed for 30 s at maximum speed. Proteinase K (40 μL) was added, and the tubes were vortexed for several seconds. Further steps followed the instructions of the manufacturer. Environmental strains of *V. cholerae* and *V. metoecus* were isolated from the August and September 2009 filters, as previously described [62].

Three different regions in Bangladesh were selected to collect environmental water samples: two coastal regions (Kuakata and Mathbaria) and an inland region (Dhaka). Collection of water samples and extraction of DNA from filters from coastal Bangladesh sites (Kuakata and Mathbaria) were done at a single time point (May 2014) using the same protocol as the Oyster Pond sampling [62]. The Dhaka samples were collected from Rampura, Dhaka, Bangladesh (Figure 1) bi-weekly from October 2015 to March 2016. Water samples (50 mL) were collected using 60 mL sterile polypropylene syringe and filtered through 0.22 μm Sterivex filters (Millipore, Burlington, MA, USA). Total DNA extraction from the biomass on the filters was done by following four consecutive steps: cell lysis and digestion, DNA extraction and DNA concentrating, and washing according to the protocol described by Wright et al. [75]. Environmental strains of *V. cholerae* were isolated from the water samples collected in Dhaka by following the protocol previously described [28].

To reduce impurities that can act as inhibitors during PCR amplification, all extracted DNA samples were further treated with One step PCR inhibitory removal kit (The Epigenetics Company, ZYMO Research, Irvine, CA, USA), following the protocol in the user manual. Treated samples were kept at −20 °C for further analysis.

### 3.3. Design and Evaluation of Primers and Fluorogenic Probes for Real–Time qPCR

To design a primer set suitable for the amplification of products that are unique to *V. cholerae* strains, protein-coding genes from a dataset of 52 *Vibrio* genomes were analyzed as outlined in Kirchberger et al. [76]. Briefly, the genes of *V. cholerae* and its close relative, *V. metoecus* were clustered into families based on 30% similarity of amino acid sequence of proteins through OrthoMCL [77]. Alignments of core gene families exclusive to *V. cholerae* were created via CLUSTALW 2.0 [78], with single gene trees constructed through RAxML 8.0 [79]. Gene sequences were further inspected to find optimal sites for non-degenerate primer/probe design. The *viuB* gene, encoding the vibriobactin utilization protein B (WP_000064348.1) for *V. cholerae* [80,81], was selected after examination of candidates. Primers (forward and reverse) and probe (Table 2) for a 77-bp product were designed using the software tool PrimerQuest from integrated DNA technologies (IDT, Coralville, IO, USA) according to supplied guidelines.

To design primers specific for *V. metoecus*, Intella (https://www.vound-software.com/) was used to analyze the unique gene contents of *V. metoecus*. Alignments of the sequence of alleles from single copy gene families present in all *V. metoecus* in the dataset were performed by using an in-house script (available upon request). The gene encoding a methyl-accepting chemotaxis protein (MCP) (EEX66169.1) was selected for the presence of ideal primer and probe sites [82], as designed using the software PrimerQuest tool from Integrated DNA technologies (IDT, Coralville, IO, USA). This gene was targeted using the probe 5′-/5Cy5/TTG TCC GTT TCG ACA CTG AAA TCA/3IAbRQSp/-3′, and forward and reverse primers, 5′-GCA GTC TCT TGC CGA AAC ACT A-3′ and 5′-ATG AAC AGC TTA TCT TGC CAT TC-3′, respectively, yielding an 81-bp product. The designed primers were tested for specificity by end point PCR with spiked water samples.

For estimation of toxigenic *V. cholerae* abundance, 106 bp of the *ctxA* gene (Table 3) was targeted as part of the genetic element encoding the major virulence factor cholera toxin, and thus one of the signature genes for toxigenic potential in *V. cholerae* [47]. In the case of the *V. cholerae* O1 serogroup, the target was a 113 bp product of the *rfbO1* gene (Table 3) as it detects explicitly *V. cholerae* belonging to the O1 serogroup, which includes the vast majority of strains responsible for past and ongoing cholera pandemics. Primers and probes for both of these genes were designed in this study to ensure compatibility of the assay. FAM, 6-carboxyfluorescein; Cy5, Cyanine 5 dye and HEX, Hexacholo-fluorescein dye were used as a reporter dye. ZEN-Iowa Black FQ and Iowa Black RQ were used as quenchers.

### 3.4. Real-Time qPCR Amplification

Dynamite qPCR Mastermix used in this study is a proprietary mix, developed and distributed by the Molecular Biology Service Unit at the University of Alberta, Edmonton, AB, Canada. It contains Tris (pH 8.3), KCl, MgCl_2_, glycerol, Tween 20, DMSO, dNTPs, ROX as a normalizing dye, and antibody inhibited *Taq* polymerase. The volume of the PCR reaction was 10 μL containing 5 μL of 2× Dynamite qPCR master mix, 1 μL of each of 500 nM primer-250 nM probe mix, 1 μL of molecular grade water and 2 μL of DNA template. Real-time quantitative PCR was performed under the following conditions: initial primer activation at 95 °C for 2 min followed by 40 cycles of 95 °C for 15 s and 60 °C for 1 min in the Illumina Eco Real-Time PCR system. The assay includes standards of known copy number and negative control with no template added to assess the potential presence of contamination (Figure 2).

### 3.5. Generation of Standard Curves and Calculation of qPCR Efficiency

Standard curves were prepared by amplifying gene sequences from corresponding reference strains for each target. For the preparation of standard curves, the *viuB*, *ctxA* and *rfbO1* genes of the pandemic *V. cholerae* El Tor O1 N16961 reference strain were used for total *V. cholerae* count, toxigenic *V. cholerae* and *V. cholerae* O1, respectively. To make the standard curve for the *V. metoecus* specific gene (*mcp*), the *V. metoecus* RC341 was used. Both strains were grown on LB agar (BD Difco, Franklin Lakes, NJ, USA) with 0.5% NaCl at 30 °C for overnight and DNA extraction was done by DNeasy Blood and Tissue Kit (QIAGEN). Specific forward and reverse primers targeting each gene of interest were used for PCR amplification (Table 3). A standard PCR protocol was followed for this amplification: 1 μL each of 10 pmol forward and reverse primer, 0.4 μL of 10 mM dNTP-Mix (ThermoFisher, Waltham, MA, USA)*,* 0.4 μL Phire Hot Start II DNA Polymerase (ThermoFisher), 4 μL of 5× Phire Buffer, 12.2 μL of molecular biology grade water and 2 μL of template DNA. The PCR reaction was performed as follows: initial denaturation at 98 °C for 30 s, followed by annealing at 55 °C for 5 s and extension 72 °C for 1 min for 35 cycles and a final extension of 72 °C for 1 min. PCR products were purified using the Wizard SV Gel and PCR Clean-up System (Promega, Madison, WI, USA). The concentrations of amplified PCR products were measured using the Quant-iT PicoGreen dsDNA Assay Kit (Molecular Probes, Eugene, OR, USA) and the Synergy H1 microplate reader (BioTek, Winooski, VT, USA).

Calculations mentioned in the Applied Biosystems Guideline [83] for creating qPCR standard curves were used for determining the mass of amplified gene templates that correspond to copy numbers of target nucleic acid sequences. A series of standards were prepared in which a gene of interest is present at 3 × 10^5^ copies, 3 × 10^4^ copies, 3 × 10^3^ copies, 3 × 10^2^ copies 30 and 3 copies per 2 μL of the template. Once prepared, the standards were stored in 100 μL aliquots at −80 °C. The standard curve was generated by plotting the log value of calculated gene copies per reaction over the quantitative cycle value (Cq) (Figure 2). The Cq is described as the cycle at which the fluorescence from amplification exceeds the background fluorescence in the MIQE guideline [60].

If a sample contains more targets, the fluorescence will be detected in earlier cycles; low Cq values represent higher initial starting copies of the target gene. The qpCR efficiency of the assay was calculated (Figure 2) using the following formula:

(1) $$\mathrm{Efficiency}=10^{\frac{-1}{\mathrm{Slope}}}\;$$

(http://efficiency.gene-quantification.info/) by Illumina Eco Real-Time PCR system software.

### 3.6. Limit of Detection (LOD) and Impact of Inhibition Testing

The LOD of the assay was determined for each of the marker genes based on the standard curve of amplified genes from reference strains (*V. cholerae* N16961 and *V. metoecus* RC341) (Figure 2). The LOD of sample (per liter of water) before filtration was calculated from the LOD of the qPCR assay. We did not evaluate the quantifiable lowest minimum number of copies in the environmental water sample before any filtration step.

To test for qPCR inhibition, we compared the Cq values for 10× dilution of treated (with One step PCR inhibitory removal kit) extracted DNA samples from study sites and spiked positive and negative samples. The differences in Cq values between diluted samples were recorded (Supplementary Figure S2).

### 3.7. Specificity Testing

The specificity of the qPCR assay was evaluated using genomic DNA from bacterial strains listed in Table 2. Non-O1 *V. cholerae* (17) from environmental sources, *ctxA* positive and negative *V. cholerae* O1 (8 and 2 isolates, respectively), from both clinical and environmental sources, and *V. metoecus* (18) from environmental sources were tested (Supplementary Table S1). Three other *Vibrio* species: *V. parahaemolyticus*, *V. vulnificus* and *V. mimicus,* were also tested, as well as two non-*Vibrio* gammaproteobacteria: *Pseudomonas aeruginosa* and *Escherichia coli*.

## 4. Conclusions

This research for the first time describes a sensitive multitarget real-time qPCR application for the simultaneous detection and quantification of *V. cholerae* and *V. metoecus* from environmental water samples. The *viuB* and *mcp* markers are specific to *V. cholerae* and *V. metoecus*, respectively, and made it possible to quantify the absolute abundance of members of these two species in DNA extracted from environmental biomass. The *ctxA* and *rfbO1* markers are specific for detection of toxigenic and O1 serogroup strains, allowing determination of the proportion of the total *V. cholerae* population represented by these strains. This is also the first fundamental study on quantification of *V. cholerae* on a significant scale in a cholera-endemic area. Although cholera has two seasonal peaks in this region, we showed that toxigenic *V. cholerae* O1 was persistent in the inland water reservoir at levels that pose a risk to human health. *V. metoecus* was not detected in this area, indicating a different geographical distribution and seasonal presence compared to that of its closest relative. The sporadic presence of *V. cholerae* O1 at a substantial proportion of the local *V. cholerae* total population in a region not endemic for cholera also highlights the wide distribution of this lineage displaying potential for the emergence of novel virulent variants. As demonstrated by the volume of samples analyzed here, this method has a throughput that is high enough to allow for the determination of the source of an outbreak and tracking its dispersal across aquatic environments or a drinking water distribution system.

## Figures and Tables

**Table 1:** Bacterial strains used to validate specificity of the primers and probes designed to detect *V. cholerae*, its toxigenic serogroup O1 and *V. metoecus.*

			Target Genes		
**Species**	**No. of Strains**	***viuB***	***ctxA***	***rfbO1***	***mcp***
*Vibrio cholerae* non O1	17	+	-	-	-
*Vibrio cholerae* O1 CTX-	2	+	-	+	-
*Vibrio cholerae* O1 CTX+	8	+	+	+	-
*Vibrio parahaemolyticus*	1	-	-	-	-
*Vibrio vulnificus*	3	-	-	-	-
*Vibrio metoecus*	18	-	-	-	+
*Vibrio mimicus*	3	-	-	-	-
*Escherichia coli*	3	-	-	-	-
*Pseudomonas aeruginosa*	3	-	-	-	-

+ indicates the target primers were amplified for all strains via qPCR; - indicates no amplification.

**Table 2:** Comparative results of the conventional culture method and developed qPCR technique used in this study.

Site	Time	Cultivation			qPCR		
		*V. cholerae*	*V. cholerae* O1	*V. metoecus*	*V. cholerae*	*V. cholerae* O1	*V. metoecus*
Kuakata	May’14	+	-	-	+	+	-
Mathbaria	May’14	+	-	-	+	-	-
Dhaka	Oct’15	+	+	-	+	+	-
	Nov’15	+	-	-	+	+	-
	Dec’15	+	-	-	+	+	-
	Jan’16	+	-	-	+	+	-
	Feb’16	+	-	-	+	+	-
	Mar’16	+	-	-	+	+	-
Oyster Pond	Jun’08	ND	ND	ND	+	-	-
	Jul’08	ND	ND	ND	+	+	-
	Aug’08	ND	ND	ND	+	+	+
	Sep’08	ND	ND	ND	+	-	+
Oyster Pond	Jun’09	ND	ND	ND	+	-	-
	Jul’09	ND	ND	ND	+	+	-
	Aug’09	+	-	+	+	+	+
	Sep’09	+	-	+	+	-	+

+ indicates samples were positive for corresponding organism; - indicates there was no isolation and/or negative by qPCR; ND indicates not done.

**Table 3:** Target genes and sequences of primers and probes used in this study.

Target Gene	Primer and Probe	Sequence (5′-3′)	Amplicon Size (bp)
*viuB*	Probe	56-FAM/TCATTTGGC/ZEN/CAGAGCATAAACCGGT/3IABkFQ	77
	Forward primer	TCGGTATTGTCTAACGGTAT	
	Reverse primer	CGATTCGTGAGGGTGATA	
*ctxA*	Probe	5Cy5/AGGACAGAGTGAGTACTTTGACCGAGG/3IAbRQSp	106
	Forward primer	CAGGTGGTCTTATGCCAAG	
	Reverse primer	CTAACAAATCCCGTCTGAGTT	
*rfbO1*	Probe	5HEX/AGAAGTGTG/ZEN/TGGGCCAGGTAAAGT/3IABkFQ	113
	Forward primer	GTAAAGCAGGATGGAAACATATTC	
	Reverse primer	TGGGCTTACAAACTCAAGTAAG	
*mcp*	Probe	5Cy5/TTGTCCGTTTCGACACTGAAAATCA/3IAbRQSp	81
	Forward primer	GCAGTCTCTTGCCGAAACACTA	
	Reverse primer	ATGAACAGCTTATCTTGCCATTC	

## Supplementary Materials

The following are available online at https://www.mdpi.com/2076-0817/9/12/1053/s1, Figure S1: Phylogeny of the pandemic generating lineage of *V. cholerae*. Figure S2: Testing of inhibition in qPCR amplification. Supplementary Figure S3: Biological replications of the quantification of *V. cholerae* along with its toxigenic and Serogroup O1 subpopulations and its close relative *V. metoecus* in Oyster Pond, MA, USA. Table S1: Bacterial strains used for validating primers and probes in this study.
