# Supplementary material for: Simultaneous Quantification of Vibrio metoecus and Vibrio cholerae with Its O1 Serogroup and Toxigenic Subpopulations in Environmental Reservoirs
Source: Pathogens. 2020 Dec 16;9(12):1053. doi: 10.3390/pathogens9121053 (PMC7766680; doi:10.3390/pathogens9121053)
Supplement: Supplementary file 1 [file pathogens-09-01053-s001.pdf]

Supplementary data

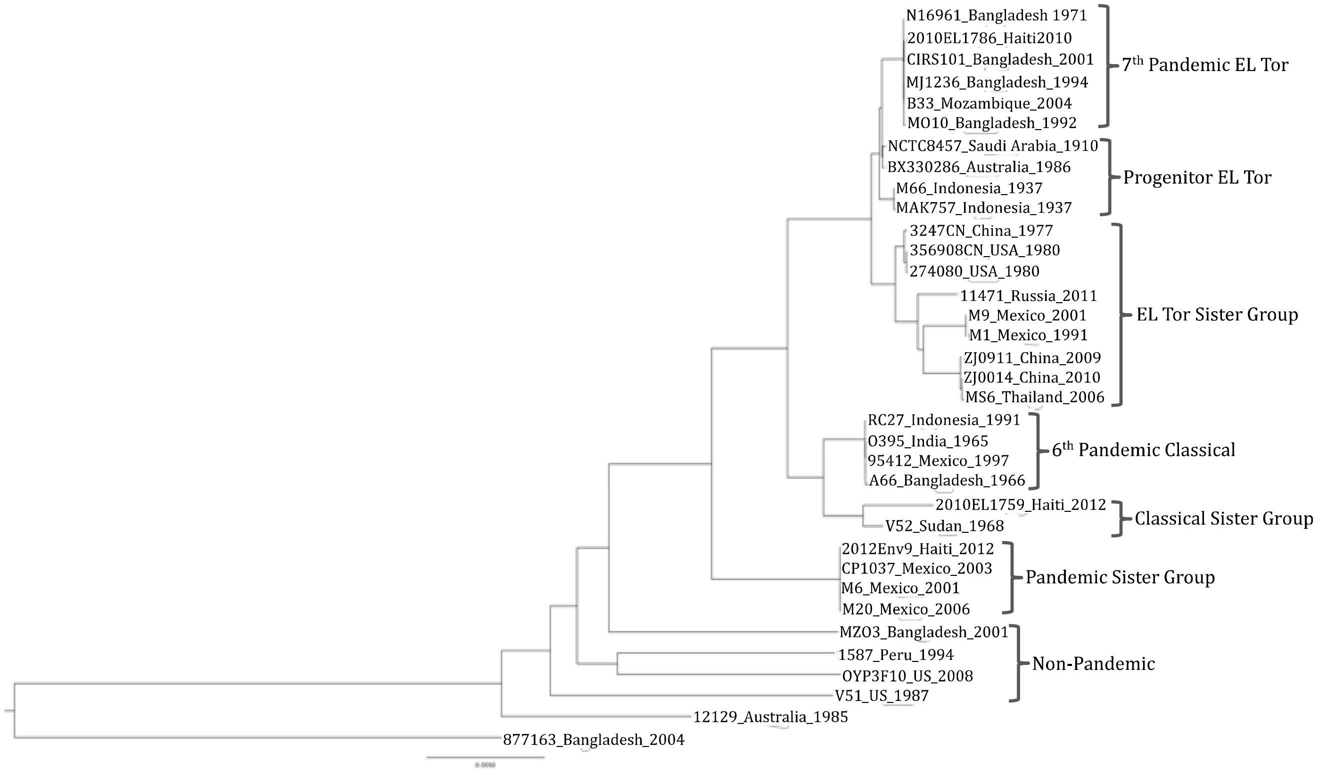

FIG S1. Phylogeny of the pandemic generating lineage of *V. cholerae*. The maximum likelihood phylogenomic tree was constructed from the alignment of locally collinear blocks (2,367,372 bp) using GTR gamma substitution model with 100 bootstrap replicates (all nodes had >98% bootstrap support). Environmental strain *Vibrio sp.* strain 877-163 was used to root the tree. The source and isolation year of the strains used are indicated next to the strain names.

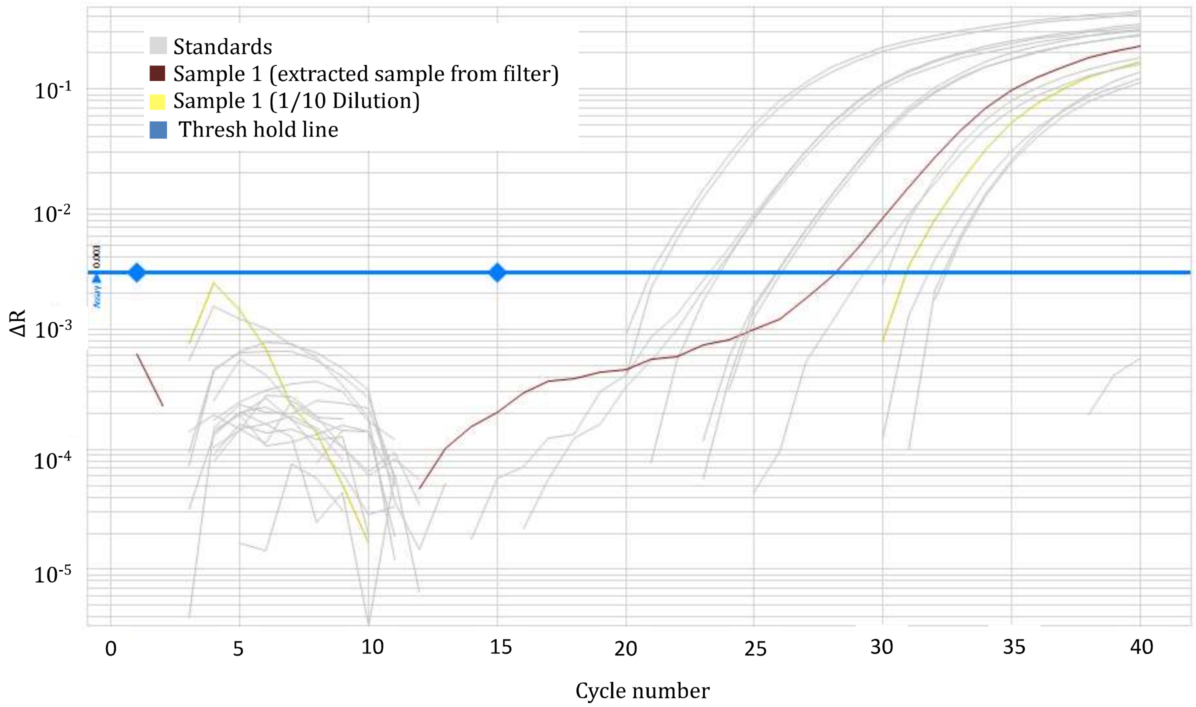

FIG S2. Testing of inhibition in qPCR amplification. The qPCR assay was performed on an extracted DNA sample treated with One step PCR inhibitory removal kit (sample1) from Oyster Pond large fraction size (> 63 µm) and a 10× dilution. Standards (3×10^4 copies to 3 copies per reaction indicated with grey colored lines) were run in the same experiment. ΔR, normalized reporter value. The 10× dilution of samples shifted the Cq values by 3.3 cycles ± 0.05, indicating no inhibition.

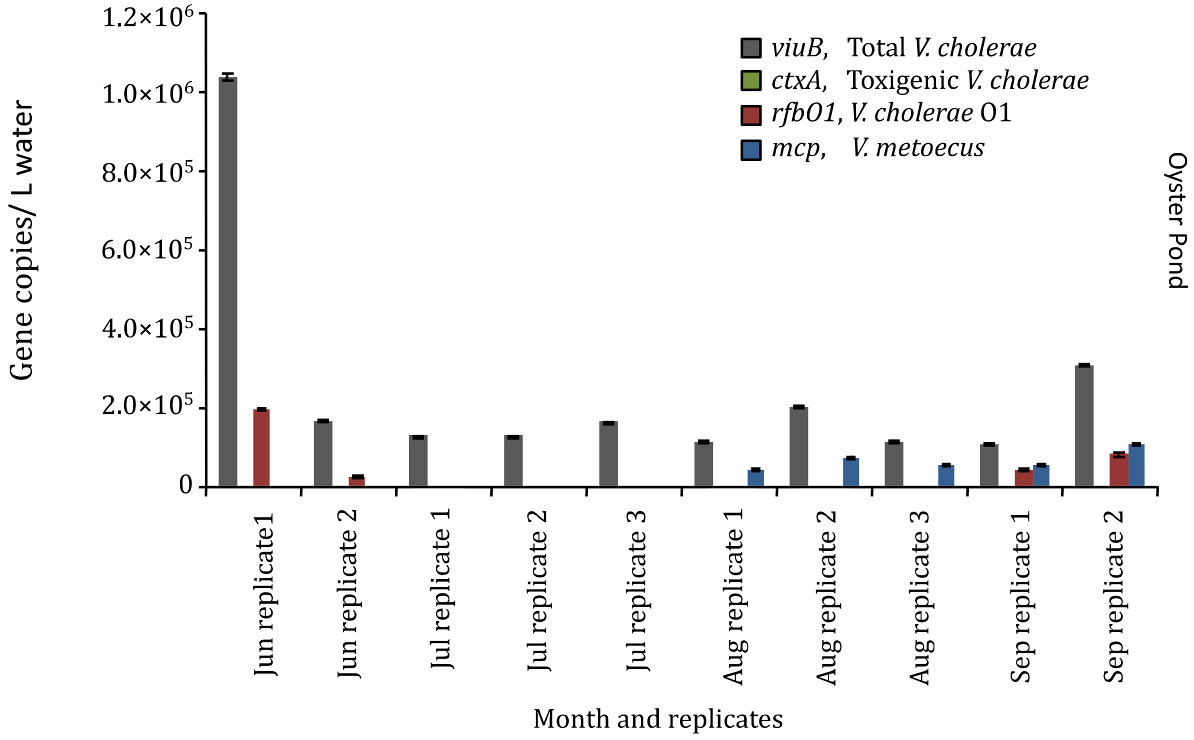

FIG S3. Biological replicates of the quantification of *V. cholerae* along with its toxigenic and serogroup O1 subpopulations and its close relative *V. metoecus* in Oyster Pond, MA, USA. Environmental water samples were collected in two or three replicates during the months of June to September in 2008. Each replicate represents 50 ml filtered independently from the same 10 L sample as its matching replicate. Bacteria were quantified by qPCR of marker genes. The *viuB* gene was used to quantify total *V. cholerae*; *ctxA* and *rfbO1* were used to measure toxigenic *V. cholerae* and *V. cholerae* O1, respectively; the abundance of *V. metoecus* was quantified using the *mcp* gene. Each qPCR reaction was run in triplicate. Mean values are shown with error bars indicating the standard deviation between technical replicates.

TABLE S1. Bacterial strains used for validating primers and probes in this study

Species	No. of strains	Strain	Classification	Accession number*
V. cholerae				
V. cholerae non O1	17	OYP1G01	Environmental	NMTO00000000
		OYP2A12	Environmental	NMTN00000000
		OYP2E01	Environmental	NMTK00000000
		OYP3B05	Environmental	LBGB00000000
		OYP3F10	Environmental	NMTJ00000000
		OYP4B01	Environmental	NMTI00000000
		OYP4C07	Environmental	LBGE00000000
		OYP4G08	Environmental	NMTH00000000
		OYP4H06	Environmental	NMTG00000000
		OYP4H11	Environmental	NMTE00000000
		OYP6D06	Environmental	NMTC00000000
		OYP6E07	Environmental	NMTB00000000
		OYP6F08	Environmental	NMTA00000000
		OYP6F10	Environmental	NMSZ00000000
		OYP7C09	Environmental	NMSX00000000
		OYP8C06	Environmental	NMSV00000000
		OYP8F12	Environmental	NMSU00000000
V. cholerae 01 CTX+	8	N16961	Clinical	AEOO3852
		V52	Clinical	AEOO3853
		EDC-728	Environmental	MIPNOOOOOOOO
		EDC-753	Environmental	
		EDC-754	Environmental	
		EDC-755	Environmental	
		EDC-772	Environmental	
		EDC-805	Environmental	
V. cholerae 01 CTX-	2	M6	Clinical	NZ_JAACMFOOOOOOOOO
		M2O	Clinical	NZ_JAACMBOOOOOOOOO

Species	No. of strains	Strain	Classification	Accession number*
Other *Vibrio* species				
V. parahaemolyticus	1	ATCC 17802	Type strain, Clinical	CP014046: CP014047
V. vulnificus	3	ATCC 27562	Type strain, Clinical	CP012882: CP012881
		CVD7	Environmental	NA
		A9	Environmental	NA
V. metoecus	18	RC341	Environmental	ACZT00000000
		OP3H	Environmental	JJMN00000000
		OYP4D01	Environmental	LBGO00000000
		OYP4E03	Environmental	NMST00000000
		OYP5B04	Environmental	LBGP00000000
		OYP5B06	Environmental	LBGQ00000000
		OYP5H08	Environmental	NMSR00000000
		OYP8G05	Environmental	NMSQ00000000
		OYP8G09	Environmental	NMSP00000000
		OYP8G12	Environmental	NMSO00000000
		OYP8H05	Environmental	NMSN00000000
		OYP9B03	Environmental	NMSM00000000
		OYP9B09	Environmental	NMSL00000000
		OYP9C12	Environmental	NMSK00000000
		OYP9D03	Environmental	LBGR00000000
		OYP9D09	Environmental	NMSJ00000000
		OYP9E03	Environmental	NMSI00000000
		OYP9E10	Environmental	NMSH00000000
V. mimicus	3	ATCC 33653	Type strain, Clinical	NA
		ATCC 33654	Type strain, Environmental	CP014042: CP014043
		ATCC 33655	Type strain, Clinical	AOMO00000000
Other bacterial species				
Escherichia coli	3	CU1	Clinical	NA
		CU2	Type strain	NA
		MSU1	Clinical	NA
Pseudomonas aeruginosa	3	12A1	Clinical	NA
		PA103	Type strain, Clinical	JARI00000000
		PA14	Clinical	CP000438

* Accession number obtained from Genebank
